# Changes in community members’ toenail metal concentrations after cessation of nearby open-burn thermal treatment of munitions waste

**DOI:** 10.1038/s41370-026-00872-9

**Published:** 2026-05-06

**Authors:** Myron L. Lard, Chuqi Guo, Qingzhao Yu, Thomas Blanchard, Brenda Vallee, Wilma Subra, Oluwafeyikemi Ogunmusi, Stephania A. Cormier, Jennifer Richmond-Bryant

**Affiliations:** 1 Department of Forestry and Environmental Resources, North Carolina State University, Raleigh, NC USA; 2 Louisisana State University Health Sciences Center, New Orleans, LA USA; 3 Department of Oceanography and Coastal Sciences, Louisiana State University and A&M College, Baton Rouge, LA USA; 4 Central Louisiana Coalition for a Clean and Healthy Environment, Colfax, LA USA; 5 Subra Company, New Iberia, LA USA; 6 Department of Environmental Sciences, Louisiana State University and A&M College, Baton Rouge, LA USA; 7 Department of Biological Sciences, Louisiana State University and A&M College, and Pennington Biomedical Research Institute, Baton Rouge, LA USA

**Keywords:** Toenail analysis, Heavy metals, Exposure assessment, Hazardous waste, Munitions, Open-burn/open-detonation thermal treatment

## Abstract

**Background:**

Colfax, LA, is a rural, predominantly minority community, where an open-burn/open-detonation (OB/OD) hazardous waste thermal treatment (TT) facility operated until December 2023.

**Objective:**

This study aimed to identify the presence of metals in the toenails of Colfax residents before and after cessation of OB/OD.

**Methods:**

Toenail samples were collected from community members in 2023–2024 (during facility operation) and 2025 (post-cessation) at locations near ( < 7.5 km) and far ( > 7.5 km) from the facility.

**Results:**

Elevated levels of heavy metals, including lead, were observed in 2023–2024, with generally lower concentrations in 2025. Statistically significant decreases were observed for molybdenum, nickel, and lead after OB/OD ceased. In the 2023–2024 collection, samples obtained closer to the facility were more likely to have elevated levels of aluminium, iron, nickel, and lead.

**SIGNIFICANCE:**

Exposure to OB/OD likely contributed to increased metal body burden among Colfax residents. Results suggest cessation of operations reduced community exposures to heavy metals.

**Impact:**

- Open-burn/open-detonation (OB/OD) hazardous waste thermal treatment poses risk of exposure to toxic contaminants, including heavy metals, environmentally persistent free radicals, dioxins, and furans. We measured changes in toenail concentrations of heavy metals while an OB/OD facility was in operation and again 15 months after cessation. Our data suggests that cessation of OB/OD results in decreased exposure and reduced body burden for some toxic metals, including lead, aluminium, iron, and nickel.

## Introduction

Colfax is a small town (pop. 1442) located in central Louisiana, with a community makeup of primarily low-income, minority residents [1]. An open-burn/open-detonation (OB/OD) thermal treatment (TT) facility, that was once responsible for the disposal of hazardous materials, including superfund site waste, fireworks and propellants, and military waste is located approximately 5 km north of northern border of the town and 1.5 km north of The Rock community, a historically Black enclave [2, 3]. Our previous research has identified the significant potential for airborne exposures in the community from the facility’s practices [4]. highlighted by air pollution plume models, resident interviews describing common health conditions arising in the community, and facility burn records as reference data. It is important to note that through community organization, supported by research efforts, the TT facility was issued a new permit in 2023, requiring them to stop OB/OD practices [3]. No reported activity has been conducted since the end of 2023.

The widespread impact of the TT practices on the surrounding community is a major area of concern, as the burning of hazardous energetic materials can lead to the generation of harmful airborne byproducts [5–9]. To identify these potential impacts, this work aimed to analyze the metal content of residents’ toenails. Previous research has used the analysis of toenails as a measure of exposure [10–15], as it has been shown that numerous metals can be excreted through the toenail [12–15]. Inductively coupled plasma (ICP) analysis was used for detecting the presence and concentration of heavy metals in toenails, which would indicate a resident’s exposure. The hazardous metals measured include lead (Pb) and antimony (Sb), which are directly connected to serious potential health risks, including cardiovascular, pulmonary, developmental, and reproductive issues [16–18]. Toenails are expected to contain metals from exposures occurring in the 7-12 months prior to the date on which they were collected [19].

We hypothesize that detectable levels of harmful metals, including lead (Pb) and nickel (Ni), were reduced in the toenail samples collected in 2025, following the expected decrease in pollution generated after the cessation of the OB/OD TT practices at the facility. To test this hypothesis, this study aims to identify the presence of metals in the toenails of Colfax residents. Additionally, we compare measured concentrations over time and distance from the facility to identify any potential influence from the TT practices that were taking place prior to December 2023.

## Methods

**Fig. 1:**
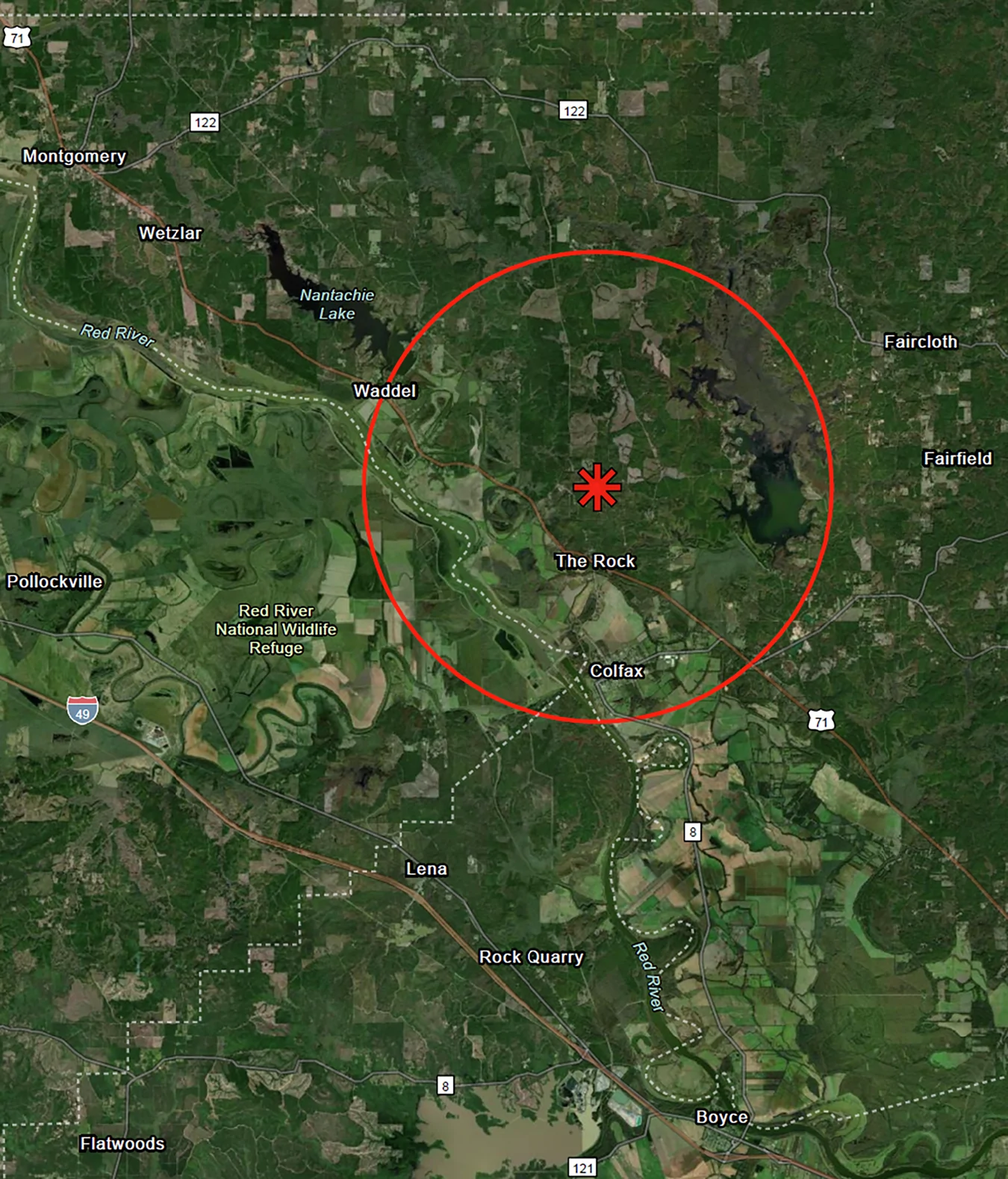
Satellite image of the sample area with the TT facility indicated by an asterisk, and a 7.5 km delineation indicated by a red circle.

Sampling took place in August 2023 and January 2024 to capture exposures to emissions from the TT facility, which ceased open burning in December 2023. Samples of toenails were collected at the homes of 52 residents (50% Black or African American, 50% White, 62% Female, 38% Male, Ages 24-89) during this time period, with 36 samples obtained within 7.5 km of the TT facility and 16 samples obtained beyond 7.5 km. The 7.5 km radial delineation was chosen in part to divide those living in The Rock and the town of Colfax from those living outside the town (Fig. 1) and is also based on preliminary analysis of the data, as discussed further below with regard to the statistical analysis. Samples were received again in March 2025, 15 months after the TT facility stopped operating.

Study participants were provided with nail polish remover pads if they wore nail polish and wanted to participate. Participants were given a fresh pair of toenail clippers and instructions to clip the toenails onto a sheet of paper that was folded and placed in a plastic Ziplock bag. The bag was labeled with a random number, which was entered into a crosswalk table that contained information about the address and demographic characteristics of the study participants. Institutional Review Board approval was granted by the North Carolina State University Office of Research on June 22, 2022 (Protocol #25124).

The toenail samples were acid digested for metal analysis by Inductively Coupled Plasma Optical Emission Spectroscopy (ICP-OES 5800, Agilent Technologies, USA). Samples were first sonicated for 1 h in 1% Triton X-100 (Millipore) to remove surface contaminants. After thorough rinsing with deionized water ( ≥ 18.2 MΩ·cm), the samples underwent a second 1-h sonication in HPLC-grade acetone (Honeywell), followed by additional rinsing with deionized water. Cleaned samples were dried at 60 °C for 18 h and equilibrated at room temperature for 30 min before weighing. Each dried toenail sample was acid digested in a precleaned Teflon vial by a mixture of 1.8 mL concentrated HNO₃ (Aristar Plus®, VWR) and 0.3 mL H₂O₂ (Aristar® Ultra, VWR). Vials were loosely capped and left overnight at room temperature for initial reaction, then sealed and heated at 110 °C for 1 h. After cooling, 0.3 mL of H₂O₂ was added. Digested samples were transferred to 25 mL volumetric flasks, diluted to volume with deionized water, mixed, and photo-oxidized (7900, ultra-violet, Ace Glass Inc.) for 12 h to remove organics. Samples were analyzed with recovery standards (indium, neodymium) and an internal standard (yttrium). The 22 metal(loid)s analyzed in this study were silver (Ag), aluminum (Al), arsenic (As), boron (B), barium (Ba), beryllium (Be), calcium (Ca), cadmium (Cd), cobalt (Co), chromium (Cr), copper (Cu), iron (Fe), magnesium (Mg), manganese (Mn), molybdenum (Mo), Ni, Pb, antimony (Sb), titanium (Ti), vanadium (V), tungsten (W), and zinc (Zn). Instrument and quality assurance/quality control procedures are detailed in Supplementary Text S1.

Several statistical analyses were performed to test for statistically significant changes between the operational period (2023–2024) and post-operation (2025). Sixteen participants were lost to follow-up, with 8 unable to be reached, 3 declining to participate further, 1 unavailable due to caregiving for family, 1 unavailable due to recovery from a medical procedure, 1 out of town at the time of follow-up, 1 moved to a different part of the state, and 1 deceased. For this reason, statistical analyses were performed both for the full study cohort and the cohort that was provided with samples in 2025.

First, summary statistics were obtained for 2023–2024 for the entire study population. The Mann–Whitney test was performed for the 36 participants with samples in 2023–2024 and 2025 to compare the distributions of data for each heavy metal. The Mann–Whitney test was performed because the sample sizes were relatively small, and within each metal, the data were not normally distributed. Given the small sample size, χ^2^ tests were performed to test if the proportion of residents living within 7.5 km of the TT facility whose toenail metal concentrations were higher than the median levels for those living outside the 7.5 km radius was statistically significant. A distance cutoff was needed to facilitate group comparison, provided by the χ^2^ tests. These tests also used only data for the 36 participants who participated in both rounds of sampling. A cutoff of 7.5 km was selected based on inspection of scatter plots of the 2023–2024 data comparing metal concentrations with distance from the TT facility. Concentration diminished at a distance of 7.5 km for most of the metals included in the analysis (Supplementary Fig. S2). Outliers were not removed from the analysis, given that our participant numbers were already low and that we had no reason to believe that elevated toenail metal concentrations were incorrect.

## Results and discussion

### Summary statistics

ICP analysis of the toenail samples collected in 2023–2024 showed detectable concentrations in 17/22 metals tested (Supplementary Table S3). The most prevalent metals measured in the residents’ toenails were Fe, Ca, and Mg, with maximum concentration values of 9270, 1859, and 288 mg/kg, respectively. While these are essential elements that mostly come from diet [20]. It is also known that metals such as Fe and Mg are used in munitions casings, frequently disposed of by OB/OD at the TT facility in Colfax [21]. Additionally, analysis revealed detectable levels of harmful heavy metals, including Sb and Pb. Any detected level of Pb is significant, as there is no safe level of lead permissible in the body without potential for health impacts [22]. Concentrations of As and Cd in the toenails sampled were below the limit of detection.

**Fig. 2:**
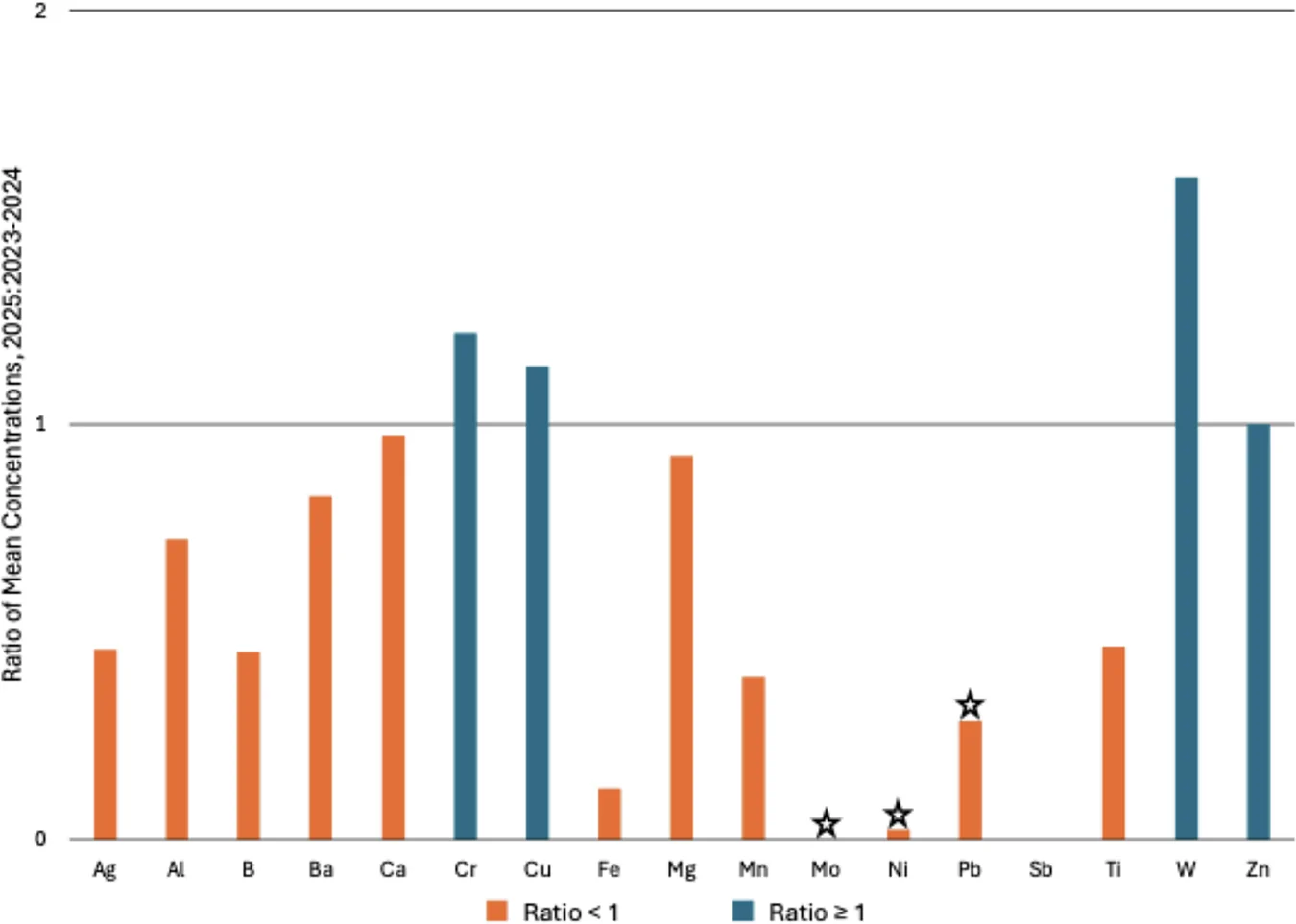
Mean concentration ratios comparing the 2025 values to those observed in 2023–2024. Stars indicate statistically significant decreases (Mann–Whitney) in concentrations between sampling periods.

Analysis of the toenails collected in the 2025 sample set (*n* = 36) showed the presence of 15/22 metals tested at varying concentrations (Supplementary Table S3). The most prevalent metals in this sample set were again Ca, Fe, and Mg, with maximum concentrations of 1697, 227, and 198 mg/kg, respectively. Concentrations of Pb were still detectable in the 2025 toenails; however, Sb concentrations were no longer present. Mo was detected in 2023–2024 but was not present in 2025. Figure 2 illustrates the ratio between the concentrations detected in 2025 and those in 2023–2024. Statistically significant decreases in concentration were seen for Mo, Ni, and Pb.

When compared to available values in the literature, the 2023–2024 samples from Colfax displayed elevated mean concentrations of numerous metals. Levels of Cr, Cu, Fe, Ni, Pb, and Zn measured in 2023–2024 were above reported mean values (1.91 ± 1.66 [23]. 5.05 ± 4.64 [23]. 41.4 ± 43.6 [24]. 32.9 ± 69.0 [23]. 0.74 ± 1.23 [23]. 120.0 ± 51.5 [24]. respectively) collected in other toenail exposure analyses conducted in the United States [13, 23, 24]. Average toenail concentrations for Al, Fe, and Ni for samples obtained in Colfax in 2025 were lower than the averages reported in previous studies [13, 23, 24].

### Temporal comparison before and after OB/OD cessation

Comparisons between the two data sets show significant differences in toenail metal concentrations for Colfax residents who provided samples in both years (*n* = 36). Of the 17 detected metals in the 2023–2024 samples, the mean concentrations of 15 metals decreased in 2025, with two metals (Sb and Mo) no longer detectable at all. Concentrations of Cr, Cu, and W increased, and concentrations of Zn remained constant between sampling campaigns. Mann–Whitney analysis revealed that decreases in mean concentrations were statistically significant for Ni, Pb, and Mo (Supplementary Table S4).

Maximum concentrations of toenail metals generally decreased for the samples collected in 2025. The maximum concentrations of several heavy metals showed substantial reductions during the sampling periods. Fe concentrations decreased more than twentyfold, from a maximum of 4811 to 277 mg/kg; and Ni concentrations decreased more than fifteenfold, from a maximum of 495 to 31.4 mg/kg. Additionally, the highest observed concentrations of Mn and Pb were reduced more than two-fold, with a 2.86x reduction for Mn and 2.06x reduction for Pb.

### Spatial comparison before and after OB/OD cessation

After analysis, the data were visualized spatially to determine any potential differences in toenail concentration as a result of distance from the TT facility. Participants’ proximity was divided into two groups: those who live within 7.5 km of the facility and those who live further than 7.5 km from the facility. Thirty-six residents lived within 7.5 km of the facility in 2023–2024, while 16 lived further than 7.5 km from the facility. For participants who provided samples in both 2023–2024 and 2025, the numbers of participants in each group were 24 ( < 7.5 km) and 16 ( > 7.5 km).

**Fig. 3:**
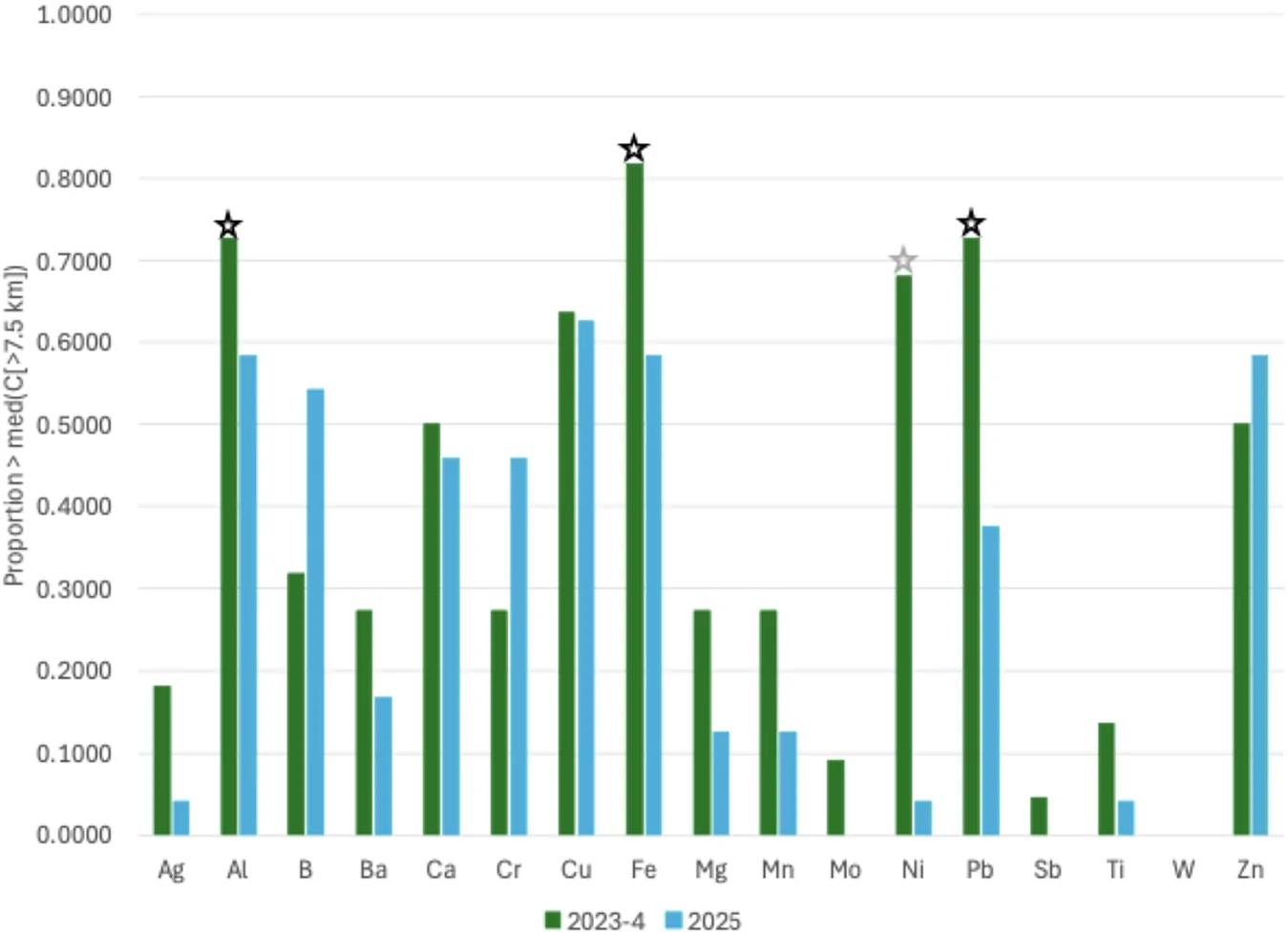
χ^2^ analysis results comparing both the temporal and spatial differences between the 2023–2024 sample and the 2025 samples for individuals participating in both sampling periods. Stars indicate near distance mean value significantly more likely to be elevated above the far distance mean value (Black stars: *p* < 0.05; Gray star: 0.05 < *p* < 0.10).

χ^2^ analysis was conducted to identify metals for which the near participants ( < 7.5 km) had concentrations higher than the median values seen on the far participants’ nails ( > 7.5 km), (Fig. 3, Supplementary Table S5). In the 2023–2024 sampling campaign, three metals were identified in which the near group had at least a 70% likelihood of having a higher value than the far group median (p < 0.05): Al (72.7%, CI: 53.0-100%), Fe (81.8%, CI: 62.6-100%), and Pb (72.7%, CI: 53.0–100%). Additionally, the higher concentration for metals Cu (63.6%, CI: 44.0-100%) and Ni (68.2%, CI: 48.4–100%) was slightly more prevalent in the near group for the 2023–2024 sampling. When comparing the near and far samples collected in 2025, the occurrence of elevated levels in the near group is generally decreased, with no metals identified as having a significant count of higher concentration. For the metals identified in 2023–2024 as having a significant likelihood of higher concentration, all were decreased in 2025: Al (58.3%, CI: 39.8–100%), Fe (58.3%, CI: 39.8–100%), and Pb (37.5%, CI: 21.6–100%).

The decreases in toenail metal concentrations observed between the two sample sets suggest that heavy-metal exposure to the participating residents was lower in the months leading up to the 2025 collection than exposures prior to the early collection. This finding coincides with the cessation of open-air TT practices conducted at the facility after December 26, 2023, as it is expected that toenails will excrete these metals 7-12 months after exposure [19]. Our findings support the hypothesis that levels of heavy metals observed in the participants’ toenails would decrease in the later sampling set. These findings strongly suggest a connection between the higher levels observed in the 2023–2024 samples and the TT practices taking place at the facility prior to 2024, highlighting the positive effect that stopping OB/OD TT likely had in reducing the heavy metal exposures to the surrounding Colfax community. From this study, removing the exposure to heavy metals in airborne pollution may have directly led to lower levels of these metals in the body.

The authors acknowledge that some limitations are present in the work as conducted. The small population in the Colfax area led to difficulties in securing participation, resulting in a limited sample size. A small sample size may introduce statistical bias or yield results that are not representative of the larger community. Statistical limitations were addressed by conducting the χ^2^ and Mann–Whitney nonparametric analyses to ensure that the data met the assumptions of the statistical tests. Recent analysis of the Sister Study has demonstrated seasonal patterns in toenail metals for 11 of the 17 metals analyzed [25]. To check if seasonality may have influenced our findings, we divided the 2023–2024 data (during or right after OB/OD) by month of acquisition to test this, and no statistically significant difference was observed between the August and January data, with the exception of Ni and Pb. Our limited sample size makes it difficult to ascertain if the nickel and lead results were related to seasonality or some other factor, but they would likely not be dietary, given that neither is an essential element, as explained in Wojcik et al. [25]. This limited sample was also not wholly representative of the demographic makeup of the Colfax community, with participants in our study being split 50:50 Black residents to White residents compared with 66:34 for the town overall [26]. These limitations were addressed by conducting our study over a broader sampling area over time, allowing for both spatial and temporal analyses. Additionally, the sampling was unintentionally split 50:50 demographically in both the near and far groups, meaning that bias due to racial differences in biological concentrations should not be observed.

## Conclusions

This work identified the presence of a number of metals in the toenails of Colfax residents. When the facility was still operating, concentrations were generally higher closer to the facility ( < 7.5 km) compared to those samples obtained further from the facility ( > 7.5 km). These higher values closer to the facility suggest some connection between the TT practices and the heavy metal concentrations seen throughout the community members’ toenails.

Metal concentrations observed in the Colfax residents’ toenails that were collected in 2025 were generally lower when compared to the earlier collected toenail samples. This is a notable finding as the TT facility halted all OB/OD practices in December 2023, removing the potential for facility emissions to influence the 2025 sample concentrations. Statistically significant decreases were observed for several metals, including Pb. These lower levels after the cessation of the OB/OD practices suggest a strong connection between the emissions generated by the facility and the elevated concentrations observed in the residents’ toenails during the 2023–2024 sampling period. The decreased levels seen in 2025 indicate lower levels of community exposure after the OB/OD was ended.

## Supplementary information

## Data Availability

Due to the personally identifiable nature of the data, deidentified data might be provided upon request, upon approval by North Carolina State University’s Institutional Review Board.

## References

[1] US Census Bureau, DP05 American Community Survey Demographic and Housing Estimates. American Community Survey 5-Year Estimates 2023.

[2] Orenczuk A. Colfax Community Speaks Up Against Clean Harbors Waste Disposal Permit Renewal, 2022.

[3] Louisiana Department of Environmental Quality (LDEQ), Complaint Results: Clean Harbors Colfax (LDEQ AI: 32096) 2024.

[4] Richmond-Bryant J, Odera M, Subra W, Vallee B, Tucker C, Oliver C, et al. A community-integrated geographic information system study of air pollution exposure impacts in Colfax, LA. Local Environ. 2022;27:728–46. 35757155 10.1080/13549839.2022.2075840 PMC9221660

[5] Dellinger B, Taylor P. Chemical aspects of combustion of hazardous wastes. Cent Eur J Public Health. 1998;6:79–87.

[6] Stanmore B. The formation of dioxins in combustion systems. Combust Flame. 2004;136:398–427.

[7] Krook J, Mårtensson A, Eklund M. Sources of heavy metal contamination in Swedish wood waste used for combustion. Waste Manag. 2006;26:158–66. 16198553 10.1016/j.wasman.2005.07.017

[8] Hasselriis F, Licata A. Analysis of heavy metal emission data from municipal waste combustion. J Hazard Mater. 1996;47:77–102.

[9] Timothy NA, Williams ET. Environmental pollution by heavy metal: an overview. Int J Environ Chem. 2019;3:72–82.

[10] Goullé J, Saussereau E, Mahieu L, Bouige D, Groenwont S, Guerbet M, et al. Application of inductively coupled plasma mass spectrometry multielement analysis in fingernail and toenail as a biomarker of metal exposure. J Anal Toxicol. 2009;33:92–8. 19239734 10.1093/jat/33.2.92

[11] Button M, Jenkin GR, Harrington CF, Watts MJ. Human toenails as a biomarker of exposure to elevated environmental arsenic. J Environ Monit. 2009;11:610–17. 19280039 10.1039/b817097e

[12] Gutiérrez-González E, García-Esquinas E, de Larrea-Baz NF, Salcedo-Bellido I, Navas-Acien A, Lope V, et al. Toenails as biomarker of exposure to essential trace metals: A review. Environ Res. 2019;179:108787. 31610392 10.1016/j.envres.2019.108787 PMC8164381

[13] Salcedo-Bellido I, Gutiérrez-González E, García-Esquinas E, de Larrea-Baz NF, Navas-Acien A, Téllez-Plaza M, et al. Toxic metals in toenails as biomarkers of exposure: a review. Environ Res. 2021;197:111028. 33753073 10.1016/j.envres.2021.111028

[14] Daniel M, Ish JL, Madrigal JM, Chang CJ, Lawrence KG, Fisher JA, et al. Residential proximity to toxic metal-emitting industrial sites and toenail metal concentrations in a United States-wide prospective cohort. Environ Res. 2024;258:119466. 38908662 10.1016/j.envres.2024.119466 PMC11323170

[15] Lin JJY, Werder E, Lawrence KG, Jackson WB 2nd, Sandler DP, et al. Residential proximity to metal-emitting industries and toenail metal concentration in the US Gulf States. Expo Health. 2024;16:1185–95. 41346371 10.1007/s12403-023-00618-0 PMC12674626

[16] Debnath B, Singh WS, Manna K. Sources and toxicological effects of lead on human health. Indian J Med Spec. 2019;10:66–71.

[17] Järup L. Hazards of heavy metal contamination. Br Med Bull. 2003;68:167–82. 14757716 10.1093/bmb/ldg032

[18] Periferakis A, Caruntu A, Periferakis AT, Scheau AE, Badarau IA, Caruntu C, et al. Availability, toxicology and medical significance of antimony. Int J Environ Res Public Health, 2022;19:4669. 10.3390/ijerph19084669 PMC9030621 35457536

[19] Grashow R, Zhang J, Fang SC, Weisskopf MG, Christiani DC, Cavallari JM. Toenail metal concentration as a biomarker of occupational welding fume exposure. J Occup Environ Hyg. 2014;11:397–405. 24372360 10.1080/15459624.2013.875182 PMC4019688

[20] Hallfrisch J, Müller DC. Does diet provide adequate amounts of calcium, iron, magnesium, and zinc in a well-educated adult population? Exp Gerontol. 1993;28:473–83. 8224043 10.1016/0531-5565(93)90072-l

[21] National Academies of Sciences and Medicine, Reassessment of the Department of Veterans Affairs Airborne Hazards and Open Burn Pit Registry. The National Academies Press; Washington:2022. 36626590

[22] Vorvolakos T, Arseniou S, Samakouri M. There is no safe threshold for lead exposure: a literature review. Psychiatriki. 2016;27:204–14. 27837574 10.22365/jpsych.2016.273.204

[23] Slotnick MJ, Nriagu JO, Johnson MM, Linder AM, Savoie KL, Jamil HJ, et al. Profiles of trace elements in toenails of Arab-Americans in the Detroit area, Michigan. Biol Trace Elem Res. 2005;107:113–26. 16217136 10.1385/BTER:107:2:113

[24] Garland M, Morris JS, Colditz GA, Stampfer MJ, Spate VL, Baskett CK, et al. Toenail trace element levels and breast cancer: a prospective study. Am J Epidemiol. 1996;144:653–60. 8823061 10.1093/oxfordjournals.aje.a008977

[25] Wojcik KM, Holle AV, O'Brien KM, White AJ, Karagas MR, Levine KE, et al. Seasonal patterns in trace elements assessed in toenails. Environ Adv. 2024;15:100496. 10.1016/j.envadv.2024.100496 PMC10883685 38405619

[26] U.S. Census Bureau. S0601 Selected Characteristics of the Total and Native Populations in the United States. 2023 American Community Survey 5-Year Estimates, 2023.

