# Supplementary material for: Changes in community members’ toenail metal concentrations after cessation of nearby open-burn thermal treatment of munitions waste
Source: J Expo Sci Environ Epidemiol. Author manuscript; Available in PMC 2026 Aug 25. (PMC13502313; doi:10.1038/s41370-026-00872-9)
Supplement: Supplementary file 1 [file NIHMS2187224-supplement-Lard_et_al_suppl.docx]

**Supplemental Materials**

Text S1. Details of the ICP-AES analysis and quality assurance/quality control

All metal analyses in this study were performed using an Agilent 5800 ICP-OES (Agilent Technologies, USA) equipped with an easy-fit 1.8 mm torch one piece and a 1.8 mm ID injector (Agilent Technologies, USA), a double-pass glass cyclonic spray chamber (Agilent Technologies, USA), and a glass Seaspray nebulizer U-series (Agilent Technologies, USA). Samples were introduced using an Agilent SPS-4 autosampler (Agilent Technologies, USA) with white/white PVC tubing for sample introduction, blue/blue for the waste drain line and yellow/orange for the 10 ppm Yttrium (Y) internal standard. Indium (In) and Neodymium (Nd) were added to the digestion vials to a final concentration of 1 ppm as method recovery standards at the beginning of the digestion process. Certified reference standards (Waters ERA, Golden CO) were run every 10 samples to ensure optimal instrument performance. A 10 ppm Yttrium internal standard was used to correct for the instrumental drifts and non-spectral interferences. Matrix spikes were analyzed every 10 samples and were within 90-110 % recovery. Method detection limits (MDL) for each metal were determined using combined methods of three times standard deviation from seven (7) replicates of spiked blanks treated the same way as the samples, and the concentration levels that corresponds to an instrument signal-to-noise ratio in the range of 3 to 5^25^.

Figure S2. Scatter plots illustrating metal concentration vs. distance for each metal.

Table S3. Summary statistics for toenail data for the 2023-2024 and 2025 sampling campaigns. Data from 2023-2024 are summarized in their entirety (n = 52) and matched to the 2025 participants (n = 36).

| **Metal** | **2023-2024 (n = 52)** |  | **2023-2024 (n = 36)** |  | **2025 (n = 36)** |  |
| --- | --- | --- | --- | --- | --- | --- |
|  | **Mean (sd)** | **(Min, max)** | **Mean (sd)** | **(Min, max)** | **Mean (sd)** | **(Min, max)** |
| Ag | 5.35 (23.0) | (0, 152) | 7.29 (27.8) | (0, 152) | 3.35 (20.1) | (0, 121) |
| Al | 21.7 (27.3) | (0, 156) | 17.3 (18.3) | (0, 70) | 12.5 (14.2) | (0, 51.7) |
| As | 0 (0) | (0, 0) | 0 (0) | (0, 0) | 0 (0) | (0, 0) |
| B | 13.6 (29.5) | (0, 132) | 15.6 (35.3) | (0, 132) | 7.04 (17.5) | (0, 92.4) |
| Ba | 1.20 (3.80) | (0, 26.2) | 0.623 (1.06) | (0, 3.93) | 0.515 (1.19) | (0, 5.56) |
| Be | 0 (0) | (0, 0) | 0 (0) | (0, 0) | 0 (0) | (0, 0) |
| Ca | 861 (369) | (217, 1859) | 864 (351) | (371,1691) | 842 (348) | (327, 1697) |
| Cd | 0 (0) | (0, 0) | 0 (0) | (0, 0) | 0 (0) | (0, 0) |
| Co | 0 (0) | (0, 0) | 0 (0) | (0, 0) | 0 (0) | (0, 0) |
| Cr | 2.61 (5.10) | (0, 26.8) | 1.86 (3.51) | (0, 14.6) | 2.27 (9.39) | (0, 56.6) |
| Cu | 7.00 (4.72) | (0.503, 25.7) | 7.19 (4.77) | (0.503, 25.7) | 8.19 (13.8) | (0, 86.3) |
| Fe | 402 (1493) | (1.67, 9270) | 296 (963) | (1.67, 4811) | 36 (48.0) | (6.69, 227) |
| Mg | 111 (44.5) | (42.7, 288) | 106 (34.3) | (71.1, 202) | 98 (44.9) | (0, 198) |
| Mn | 2.46 (7.83) | (0, 53.1) | 1.12 (2.37) | (0, 10.5) | 0.436 (0.904) | (0, 3.67) |
| Mo | 0.177 (0.516) | (0, 1.86) | 0.215 (0.561) | (0, 1.86) | 0 (0) | (0, 0) |
| Ni | 41.4 (130) | (0, 720) | 37.7 (104) | (0, 495) | 0.871 (5.22) | (0, 31.4) |
| Pb | 3.44 (6.04) | (0, 33.2) | 3.65 (6.32) | (0, 33.2) | 1.04 (2.71) | (0, 16.1) |
| Sb | 0.0687 (0.491) | (0, 3.50) | 0.103 (0.601) | (0, 3.50) | 0 (0) | (0, 0) |
| Ti | 0.370 (1.29) | (0, 7.32) | 0.152 (0.519) | (0, 2.42) | 0.0708 (0.425) | (0, 2.55) |
| V | 0 (0) | (0, 0) | 0 (0) | (0, 0) | 0 (0) | (0, 0) |
| W | 1.13 (4.57) | (0, 20.0) | 0.588 (3.43) | (0, 20.0) | 0.939 (5.63) | (0, 33.8) |
| Zn | 149 (44.4) | (93.6, 255) | 140 (38.0) | (98.4, 243) | 140 (94.3) | (0, 640) |

Table S4. Mann-Whitney results for each metal, comparing matched participants from 2023-2024 and 2025 (n = 36). Statistically significant declines in mean concentration between 2024 and 2025 are shown in black bold italics (p<0.05). Test results are not provided for metals in which the concentrations in 2023-2024 and 2025 were all zero. 

| **Metal** | **p-value** |
| --- | --- |
| Ag | 0.180 |
| Al | 0.179 |
| B | 0.426 |
| Ba | 0.542 |
| Ca | 0.820 |
| Cr | 0.388 |
| Cu | 0.610 |
| Fe | 0.115 |
| Mg | 0.898 |
| Mn | 0.244 |
| ***Mo*** | ***0.0220*** |
| ***Ni*** | ***2.13 x 10***^***-5***^ |
| ***Pb*** | ***0.00841*** |
| Sb | 0.332 |
| Ti | 0.0817 |
| W | 1 |
| Zn | 0.699 |

Table S5. χ^2^ results testing if the proportion of people living within 7.5 km of the TT facility with metal concentration more than the median of those living more than 7.5 km from the TT facility was significantly greater than for the proportion of people living further than 7.5 km away, for each metal. Data from 2023-2024 are summarized in their entirety (n = 52) and matched to the 2025 participants (n = 36). 

| **Metal** | **2023-2024 (n = 51)** |  |  | **2023-2024 (n = 36)** |  |  | **2025 (n = 36)** |  |  |
| --- | --- | --- | --- | --- | --- | --- | --- | --- | --- |
|  | χ^**2**^ | **Estimate (CI)** | **p-value** | χ^**2**^ | **Estimate (CI)** | **p-value** | χ^**2**^ | **Estimate (CI)** | **p-value** |
| Ag | 15.6 | 0.147 (0.0635,1.00) | 1.00 | 7.68 | 0.182 (0.0698,1.00) | 0.997 | 18.4 | 0.0417 (0.00287,1.00) | 1.00 |
| Al | ***10.6*** | ***0.794 (0.644, 1.00)*** | ***5.60 x 10***^***-4***^ | ***3.68*** | ***0.727 (0.530.1.00)*** | ***0.0275*** | 0.375 | 0.583 (0.398,1.00) | 0.270 |
| As | 32.0 | 0.000 (0.000,0.000) | 1.00 | 20.0 | 0.000 (0.000,0.000) | 1.00 | 22.0 | 0.000 (0.000,0.000) | 1.00 |
| B | 1.44 | 0.382 (0.246,1.00) | 0.885 | 2.23 | 0.318 (0.165,1.00) | 0.932 | 0.0417 | 0.542 (0.360,1.00) | 0.419 |
| Ba | 2.38 | 0.353 (0.221,1.00) | 0.939 | 3.68 | 0.273 (0.132,1.00) | 0.972 | 9.38 | 0.167 (0.0638,1.00) | 0.999 |
| Be | 32.0 | 0.000 (0.000,0.000) | 1.00 | 20.0 | 0.000 (0.000,0.000) | 1.00 | 22.0 | 0.000 (0.000,0.000) | 1.00 |
| Ca | ***4.97*** | ***0.706 (0.551,1.00)*** | ***0.0129*** | 0.00 | 0.500 (0.335, 1.00) | 0.500 | 0.042 | 0.458 (0.286,1.00) | 0.581 |
| Cd | 32.0 | 0.000 (0.000,0.000) | 1.00 | 20.0 | 0.000 (0.000,0.000) | 1.00 | 22.042 | 0.000 (0.000,0.000) | 1.00 |
| Co | 32.0 | 0.000 (0.000,0.000) | 1.00 | 20.0 | 0.000 (0.000,0.000) | 1.00 | 22.042 | 0.000 (0.000,0.000) | 1.00 |
| Cr | 4.97 | 0.294 (0.173,1.00) | 0.987 | 3.68 | 0.273 (0.132,1.00) | 0.972 | 0.0417 | 0.458 (0.286,1.00) | 0.581 |
| Cu | 0.0294 | 0.529 (0.378,1.00) | 0.432 | 1.14 | 0.636 (0.440,1.00) | 0.143 | 1.04 | 0.625 (0.438,1.00) | 0.154 |
| Fe | ***15.6*** | ***0.853 (0.710,1.00)*** | ***4.00 x 10***^***-5***^ | ***7.68*** | ***0.818 (0.626,1.00)*** | ***0.00279*** | 0.375 | 0.583 (0.398,1.00) | 0.270 |
| Mg | 0.735 | 0.412 (0.272, 1.00) | 0.804 | 3.68 | 0.273 (0.132,1.00) | 0.972 | 12.0 | 0.125 (0.0391,1.00) | 1.00 |
| Mn | 0.735 | 0.412 (0.272, 1.00) | 0.804 | 3.68 | 0.273 (0.132,1.00) | 0.972 | 12.0 | 0.125 (0.0391,1.00) | 1.00 |
| Mo | 24.7 | 0.059 (0.0126,1.00) | 1.00 | 13.1 | 0.0909 (0.0196,1.00) | 1.00 | 22.0 | 0.000 (0.000,0.000) | 1.00 |
| Ni | 1.44 | 0.618 (0.462,1.00) | 0.115 | 2.23 | 0.682 (0.484,1.00) | 0.0678 | 18.4 | 0.0417 (0.00287,1.00) | 1.00 |
| Pb | ***15.6*** | ***0.853 (0.710,1.00)*** | ***4.00 x 10***^***-5***^ | ***3.68*** | ***0.727 (0.530,1.00)*** | ***0.0275*** | 1.04 | 0.375 (0.216,1.00) | 0.846 |
| Sb | 28.3 | 0.029 (0.00202,1.00) | 1.00 | 16.4 | 0.0455 (0.00313,1.00) | 1.00 | 22.0 | 0.000 (0.000,0.000) | 1.00 |
| Ti | 13.0 | 0.177 (0.0837,1.00) | 1.00 | 10.2 | 0.136 (0.0427,1.00) | 0.999 | 18.4 | 0.0417 (0.00287,1.00) | 1.00 |
| V | 32.0 | 0.000 (0.000, 0.000) | 1.00 | 20.0 | 0.000 (0.000,0.000) | 1.00 | 22.0 | 0.000 (0.000,0.000) | 1.00 |
| W | 28.3 | 0.0294 (0.00202,1.00) | 1.00 | 20.0 | 0.000 (0.000,0.000) | 1.00 | 22.0 | 0.000 (0.000,0.000) | 1.00 |
| Zn | 0.265 | 0.559 (0.406,1.00) | 0.303 | 0.000 | 0.500 (0.335,1.00) | 0.500 | 0.375 | 0.583 (0.398,1.00) | 0.270 |
